# How does maternal age affect genomic stability in the offspring?

**DOI:** 10.1111/acel.13612

**Published:** 2022-04-15

**Authors:** Ádám Sturm, Tibor Vellai

**Affiliations:** ^1^ Department of Genetics Eötvös Loránd University (ELTE) Budapest Hungary; ^2^ ELKH‐ELTE Genetics Research Group Budapest Hungary

**Keywords:** 5‐methylcytosine, aging, epigenetic reprogramming, genomic instability, maternal age, transposon

## Abstract

In high‐income countries, women tend to give birth at increasingly advanced ages. Despite its physiological, developmental, and medical consequences, why this tendency significantly affects genetic stability of the offspring remains largely unresolved. Accumulating evidence indicates that the higher the age of the mother at fertilization, the more intense the activity of transposable elements causing insertional mutations in functional DNA stretches in her oocyte involved in zygote formation.

Chromatin is a complex of DNA and histone proteins that forms chromosomes. Its constitution can change at genetic (alterations in DNA sequence) and epigenetic (diverse chemical modifications without affecting the DNA sequence) levels, and both alterations can be inherited to the daughter cells and the offspring, in which these variations often interfere with gene activity. Methylation is the most common epigenetic change that modifies the structure of DNA, transforming specifically cytosine and adenine nucleobases. It primarily converts cytosine to 5‐methylcytosine (5mC) and adenine to *N^6^*‐methyladenine (6mA). In general, 5mC has a role in gene repression, including X chromosome inactivation, genomic imprinting, and silencing transposable elements (TEs), whereas 6mA is associated with gene expression (Deniz et al., 2019).

During early development in mammals, two epigenetic reprogramming waves take place to eliminate established epigenetic patterns determining cell lineage and to restore pluripotent potential. One of these waves occurs in the soma, while the other does in the germ line. These epigenetic transitions include the elimination of the original DNA methylation pattern (as part of the erasure of epigenetic memory) and the creation of a new DNA methylation pattern (as part of the restoration of epigenome). The latter process is called *de novo* DNA methylation and generates predominantly 5mC marks (Greenberg & Bourc'his, 2019; Sendžikaitė & Kelsey, 2019). It is intriguing that there is a significant difference between females and males in the *de novo* DNA methylation processes, especially in the germ line that produces terminally differentiated gametes, oocytes, or sperms (Figure 1).

**FIGURE 1:**
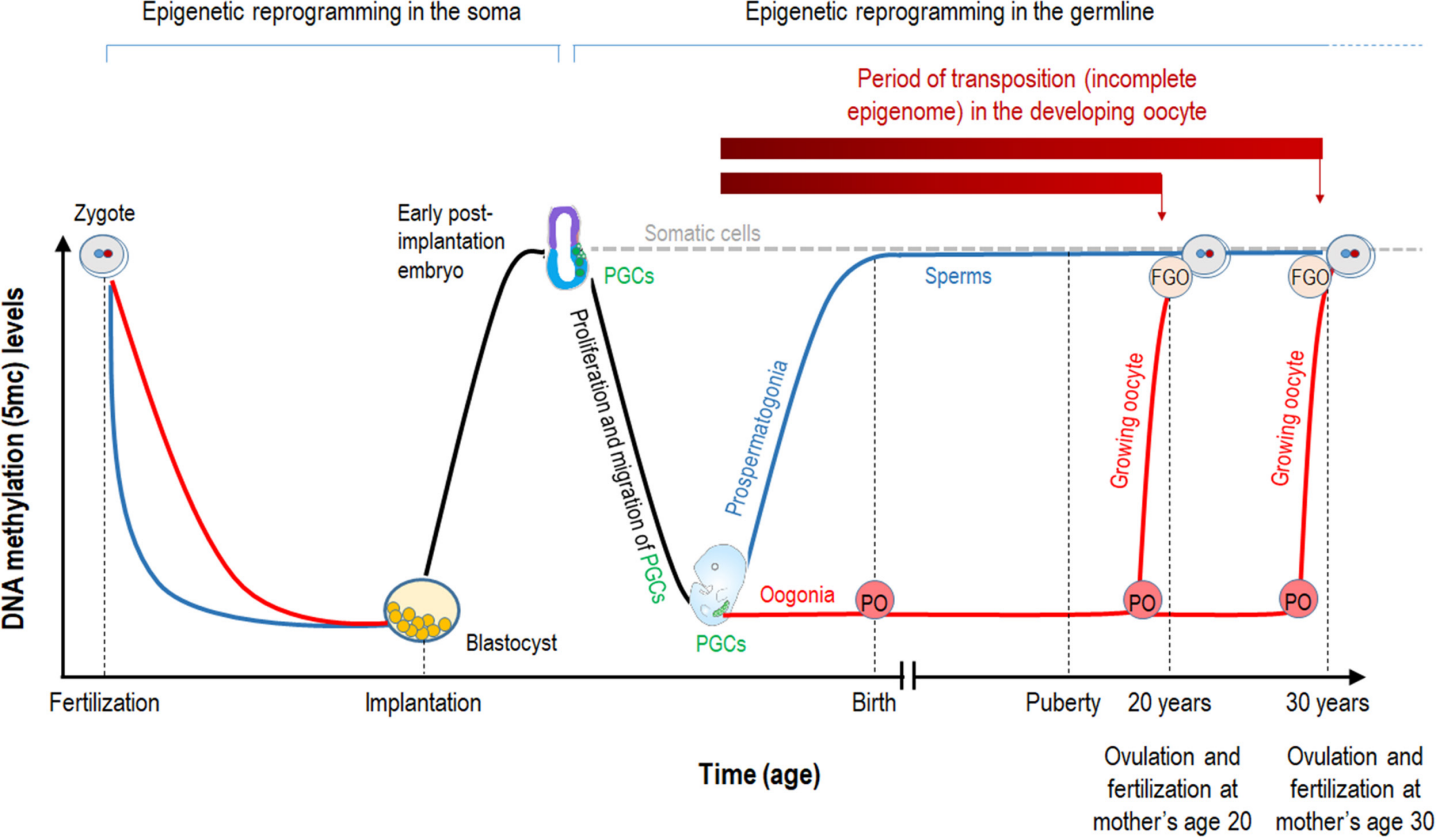
Period of *de novo* DNA methylation in the growing oocyte affects genomic integrity in the offspring. Scheme of the DNA demethylation and *de novo* DNA methylation processes in humans. Cells of the early embryo undergo a wave of DNA demethylation to eliminate gametic epigenome (erasure). The process is faster in the paternal genome (blue) than in the maternal genome (red). A new 5‐methylcytosine (5mC) pattern is then established in the early post‐implantation embryo, starting from the stage of blastocyst implantation. In the somatic cells, 5mC levels remain nearly stable for the rest of life. The germ line arises from somatic cells of the post‐implantation embryo. The emerged primordial germ cells (PGCs) proliferate and migrate to the genital ridge, then differentiate into prospermatogonia (in males) or oogonia (in females). The new somatic epigenome is erased from PGCs during these processes. Gamete‐specific epigenomes are established in the germ line by birth in men and in adulthood in women. Oogonia divide and arrest cell cycle at the first meiotic prophase stage. By the end of the fetal period, all non‐growing primary oocytes (POs) are formed and maintained in the ovary until puberty. Upon puberty, a few (15–20) POs are recruited during each menstrual cycle, and only one of them matures into a fully grown oocyte (FGO), which is eventually ovulated. During this growth process, the oocyte finishes meiosis I and is arrested again at the meiotic metaphase II stage. Its cell cycle terminates after fertilization only. *De novo* DNA methylation is completed when the oocyte enters the preovulatory stage, following growth. Until this stage, 5mC deposits are not fully restored on transposable elements, making the occurrence of transposition possible. Thus, the transposition rate in a given oocyte depends on maternal age. The younger the mother's age at fertilization, the lower the mutagenic (transposition) load in the oocyte participating in fertilization

Following fertilization, embryonic cells undergo DNA demethylation to erase the gametic epigenome and regain totipotency. This process is distinct on the parental genomes; DNA demethylation of the paternal genome is rapid and active (enzymatically mediated), whereas DNA demethylation of the maternal genome is slower and passive (DNA replication‐dependent) (Sendžikaitė & Kelsey, 2019). Then, starting at the stage of blastocyst implantation, a new DNA methylation pattern is rapidly established in both sexes, which is largely preserved in somatic cells during life span. However, in embryos at the stage of primordial germ cell (PGC) generation, a second wave of DNA demethylation begins in these germ‐line precursor cells. After the process is completed, gamete‐specific DNA methylation landscapes begin to be created in a sex‐specific manner (Figure 1). In mitotically arrested prospermatogonial cells of the male embryo, which arise from PGCs and differentiate into spermatogonia, *de novo* DNA methylation is completed before birth. By contrast, in the female germ line, the genome of primary oocytes, descendants of the PGC–oogonia cell lineage, remains largely unmethylated during embryogenesis, and *de novo* DNA methylation commences only after puberty in the growing oocytes. In humans, the process is accomplished just at the preovulatory stage when the oocyte is fully grown (Figure 1). Why is the asymmetric pattern of the *de novo* DNA methylation process in the germ line of the two sexes so significant?

In the past, women often gave birth during early adulthood. Their first child was generally born not long after their menarche, before age 20. Such children developed from an oocyte that had a partly open chromatin structure for nearly 2 decades (from the unmethylated PGC embryonic stage to the preovulatory stage; Figure 1) because *de novo* DNA methylation in the oocyte is completed following oocyte growth and maturation, just before ovulation (Virant‐Klun, 2015). Today, however, when women frequently become mothers well over age 30, this partly open chromatin period of the oocyte participating in fertilization extends to 3 decades or even longer (Figure 1). During this critical period, reduced levels of 5mC marks in the growing oocyte are unable to repress TEs completely (most of the 5mC in mammalian DNA resides in TE sequences; Yoder et al., 1997). By moving from a chromosomal locus to another one, active TEs can cause insertional mutations in functional—coding or regulatory—sequences, thereby leading to a significant level of genomic instability, which is a hallmark of essentially all aging cells (López‐Otín et al., 2013), and, as a consequence, defects in cellular functions. This mutagenic process frequently terminates in the senescence and subsequent death of the affected cell (Gorbunova et al., 2021; Sturm et al., 2017). Therefore, a mature oocyte in a woman at age 30 contains at one and a half times the new TEs relative to that of a woman aged 20. If transposition is self‐reinforcing, as it occurs in the case of self‐replicating retrotransposons, this mutagenesis rate could even be much higher. The effect is so significant that severe pathologies, such as those caused by chromosome aberrations (*e*.*g*., Down syndrome), happen in the progeny increasingly with maternal age.

Contrary to what happens in women, *de novo* DNA methylation in the male germ line is completed during embryogenesis, thereby conferring an essentially closed (transcriptionally silent) chromatin structure at TE loci of germ cells (Figure 1). Furthermore, in the male germ line, TEs are also inhibited by the Piwi‐piRNA (P‐element‐induced wimpy testis in *Drosophila*—Piwi‐interacting RNA) pathway at both transcriptionally and post‐transcriptionally, and this repression likewise persists throughout adulthood (Li et al., 2020). So, an adult man produces sperms with an essentially stable genome throughout the entirety of adulthood. This can explain why men can produce a healthy child even at very advanced ages.

Considering these facts, TE silencing plays a major role in maintaining genetic stability of the germ line. Due to a late stage of *de novo* DNA methylation and functional absence or aging‐related functional reduction in the Piwi‐piRNA pathway in the human developing oocyte, germ cells of women are likely to particularly prone to transposition, as it has been demonstrated in *Drosophila* and mice (Lin et al., 2020; Tharp et al., 2020—note that in mice, the continuous erosion of heterochromatin in oogonia occurs already after birth). The amount of new transposition events in an oocyte may simply correlate with the mother's age at which the ovulation of the given oocyte takes place. Together, the genome of an oocyte participating in fertilization is less stable in an older mother than in a younger one.

## AUTHOR CONTRIBUTION

A.S. contributed to the formulation of the model found in the manuscript. T.V. contributed to the formulation of the model found in the manuscript and wrote the manuscript.

## CONFLICT OF INTEREST

The authors declare no competing interest.

## Data Availability

Every data related to the manuscript is available in the manuscript.
